# Vesical Absorption

**Published:** 1869-05

**Authors:** 


### Vesical Absorption.

A correspondent of the *British Medical Journal*, in a communication to that paper, remarks:

"That the bladder is able, under special circumstances, to absorb the water from the urine contained in it, I have no doubt, from an observation made in my own person. Some years ago, I started one morning for a walk of many miles along the sea-coast, and when near my destination I was about to stop to pass urine, when I discovered to my consternation that my progress farther was arrested by the jutting rocks. My attention was immediately diverted to my novel position, and for some time I was engaged in various schemes for my extrication. As none were feasible, I was forced to remain an exile on the shore until the morrow allowed me to retrace my steps. It was near midnight when I suddenly recollected that I had been arrested almost in the act of micturition, and I thereupon emptied my bladder, but it was more from the idea of fulfilling a forgotten engagement than from necessity. My surprise was then great when I remarked that the quantity of urine was small, as I was sure, from my own feelings, that the bladder had some hours before been full. The physiological fact of absorption of the urine, or, at least, of its aqueous portion, forced itself upon my conviction, and I have not the slightest doubt that this did take place. I should state that, having no food, my hunger was great and my thirst painfully distressing; now, if the sense of thirst be due to the want of water in the system, its requirements were considerable in my case. Of course, I cannot prove to demonstration what amount of urine my bladder held at three o'clock, and what amount at eleven, but I know that the quantity was small at the latter time, and at the former my desire to micturate was as usual after having had no relief since the early morning. I believe, also, that I was the subject of another interesting physiological experiment: that my thirst was subsequently much alleviated by absorption of water from my wet clothes."

The editor of the *Journal*, commenting on this statement, says: "This subject is of much therapeutical as well as clinical interest, and especially in relation to morphia injections into the bladder in cystitis. Very different conclusions have been published as to the therapeutical effect of these injections. Dr. Braxton Hicks has found them useful; Sir Henry Thompson, useless. In a *résumé* in the *Gazette des Hôpitaux*, March 7, 1868, M. Segalas is described as admitting the absorption, as proved by experiments on animals; M. Demarquay, as finding it very feeble; MM. Russ and Susini, as denying it altogether, from experiments on healthy men. M. Edward Alling reports, in the *Bulletin Général de Thérapeutique*, six cases in which morphia injections, given in rebellious and severe cases, afforded great relief. Like Dr. Hicks, who has found them useful, he recommends full doses of the morphia; and he dissolves the salt in even a smaller quantity of water—thirty to forty drops of a solution, of which each drop contains two *milligrammes* of the salt."—*N. Y. Medical Journal.*

